# Recent and current advances in PET/CT imaging in the field of predicting epidermal growth factor receptor mutations in non-small cell lung cancer

**DOI:** 10.3389/fonc.2022.879341

**Published:** 2022-10-06

**Authors:** Na Hu, Gang Yan, Yuhui Wu, Li Wang, Yang Wang, Yining Xiang, Pinggui Lei, Peng Luo

**Affiliations:** ^1^ Department of Radiology, The Affiliated Hospital of Guizhou Medical University, Guiyang, China; ^2^ Department of Nuclear Medicine, The Affiliated Hospital of Guizhou Medical University, Guiyang, China; ^3^ School of Nursing, Guizhou Medical University, Guiyang, China; ^4^ Department of Pathology, The Affiliated Hospital of Guizhou Medical University, Guiyang, China; ^5^ School of Public Health, Guizhou Medical University, Guiyang, China

**Keywords:** PET/CT, prediction model, epidermal growth factor receptor, non-small cell lung cancer, radiogenomics

## Abstract

Tyrosine kinase inhibitors (TKIs) are a significant treatment strategy for the management of non-small cell lung cancer (NSCLC) with epidermal growth factor receptor (EGFR) mutation status. Currently, EGFR mutation status is established based on tumor tissue acquired by biopsy or resection, so there is a compelling need to develop non-invasive, rapid, and accurate gene mutation detection methods. Non-invasive molecular imaging, such as positron emission tomography/computed tomography (PET/CT), has been widely applied to obtain the tumor molecular and genomic features for NSCLC treatment. Recent studies have shown that PET/CT can precisely quantify EGFR mutation status in NSCLC patients for precision therapy. This review article discusses PET/CT advances in predicting EGFR mutation status in NSCLC and their clinical usefulness.

## 1 Introduction

Lung cancer has the highest incidence and mortality worldwide (1), with non-small cell lung cancer (NSCLC) accounting for approximately 85% of all lung cancer cases and adenocarcinoma (ADC) being the most prevalent pathological type (2). The emergence of targeted therapy of epidermal growth factor receptor (EGFR) tyrosine kinase inhibitor (TKI) paradigms has radically changed advanced NSCLC treatment and improved patient survival rates, especially for advanced lung adenocarcinoma (3). Accurate and rapid quantification of EGFR mutation status in NSCLC patients is crucial to selecting the most effective management strategy for individualized therapy and precision medicine to improve patient prognosis.

The gold standard assessment of EGFR mutation status is based on tumor tissue acquired by fine-needle aspiration, biopsy, or resection (4). However, acquiring a representative biopsy is not necessarily feasible with inherent limitations, including sampling bias due to the intratumoral heterogeneous tissue samples that are not readily available, and the invasive methods have low repeatability, may cause patient discomfort, and are time-consuming and costly, with inadequate samples or poor-quality tissue samples leading to inconclusive results (5). Despite liquid biopsy’s convenience, rapidity, and affordability, its sensitivity and stability are not ideal (6). Therefore, it is critical to develop a high-throughput and ideally non-invasive longitudinal method for EGFR mutation detection in NSCLC.

Image-based phenotyping is a promising clinical method for precision medicine, as it provides a non-invasive approach to visualizing tumor phenotypic characteristics (7). CT imaging combined with clinical characteristics has been systematically analyzed to predict EGFR mutations in NSCLC (8), with positron emission tomography/computed tomography (PET/CT) now widely applied to assess NSCLC patients undergoing targeted treatment. PET images capture the molecular tumor phenotypes indicating somatic mutations (9); thus, there is increasing interest in whether PET/CT can predict EGFR mutation status in NSCLC patients to develop individualized treatment. This review article discusses PET/CT advances in predicting EGFR mutation status in NSCLC and their clinical usefulness.

## 2 Association of ^18^F-FDG uptake PET/CT with epidermal growth factor receptor mutation status in non-small cell lung cancer

**Figure 1:**
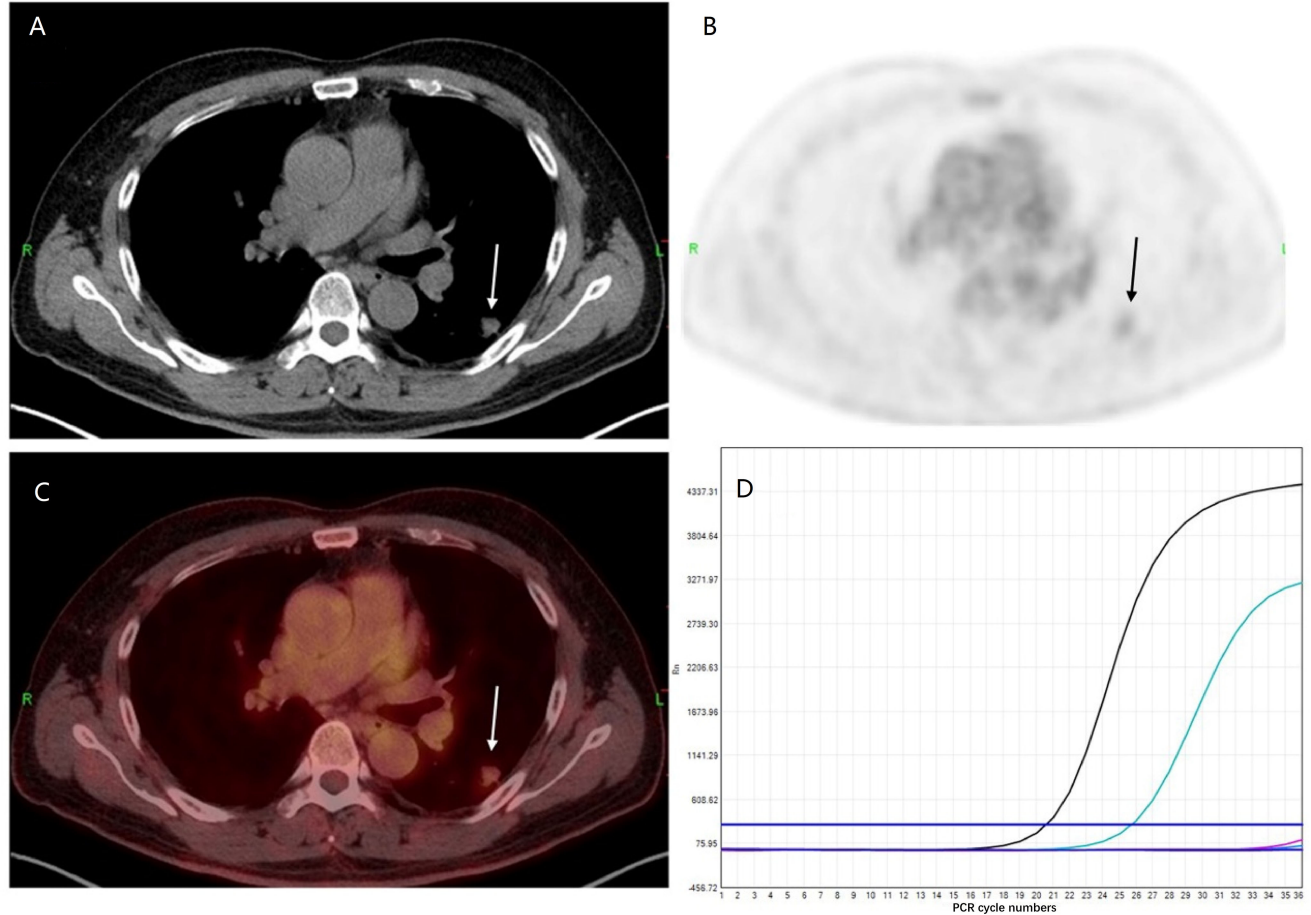
Representative epidermal growth factor receptor (EGFR) status and ^18^F-FDG PET/CT finding. A 53-year-old man with EGFR wild-type lung adenocarcinoma. **(A)** CT, **(B)** PET, and **(C)** PET/CT fusion images show a 1.0-cm-sized mild ^18^F-FDG uptake mass in the dorsal segment of the left lower lobe (SUVmax = 2.3) (arrow). **(D)** Genetic testing demonstrates wild-type EGFR status.

The EGFR signaling pathway maintains aerobic glycolysis in EGFR-mutated lung cancer cells, and EGFR TKIs have an early and profound influence on aerobic glycolysis, as they activate and promote increased oxidative phosphorylation (10), consequently indicating that EGFR mutation status is closely related to glucose metabolism in lung cancer cells. ^18^F-FDG PET/CT is increasingly used for cancer diagnosis and image-guided therapy, as it can characterize tumor cell proliferation and glucose metabolism. Accordingly, ^18^F-FDG metabolic parameters, for instance, maximum standardized uptake value (SUVmax), total lesion glycolysis (TLG), and metabolic tumor volume (MTV) may, in part, reflect EGFR mutation status in NSCLC. Numerous studies have assessed the association between ^18^F-FDG uptake and EGFR mutation status in NSCLC (**Figure 1**) but have conflicting results (**Table 1**).

**Table 1:** Recent publications about the association of ^18^F-FDG metabolic parameters of PET/CT with epidermal growth factor receptor mutation status in non-small cell lung cancer.

Authors	No. of patients	Aspect evaluated	Main results
Na et al.	100	SUVmax	A low SUVmax were more likely to possess EGFR mutation compared with patients with a high SUVmax.
Mak et al.	100	SUVmax	High FDG avidity in the primary tumor was associated with a very low chance of harboring an EGFR mutation.
Usuda et al.	148	CT imaging features and SUVmax	The EGFR mutation was significantly associated with pure or mixed GGO, lower SUVmax, and smaller tumor diameter.
Qiang et al.	97	SUVmax	Lower SUVmax was significantly correlated with the EGFR mutation group.
Guan et al.	360	SUVmax	Lower SUVmax values (SUVmax ≤ 8.1) were significantly associated with EGFR mutations.
Chen et al.	157	SUVmax	The SUVmax values were significantly lower in patients with EGFR mutations compared with patients with wild-type EGFR.
Takamochi et al.	734	SUVmax	EGFR mutations were more frequent in tumors with lower SUVmax.
Lv et al.	849	pSUVmax, nSUVmax, and mSUVmax	Low pSUVmax, nSUVmax, and mSUVmax were significantly associated with EGFR mutations.
Gu et al.	210	CEA, CT imaging features, and SUVmax	Higher CEA levels (CEA ≥ 7.0 ng/ml) and lower SUVmax (SUVmax < 9.0) were significant predictors of EGFR mutations.
Zhu et al.	139	SUVmax, SUVmean, SUVpeak, and SUVratio	SUVmax, SUVmean, SUVpeak, and SUVratio were lower in EGFR-mutated than in wild-type tumors.
Ko et al.	132	CEA, CT imaging features, and SUVmax	High SUVmax, CEA levels, and a non-spiculated tumor margin were independent predictors of the EGFR mutation.
Kanmaz et al.	218	TTF-1 and SUVmax	High SUVmax was positively correlated with EGFR mutation.
Caicedo et a	102	SUVpeak, SUVmax, and SUVmean	No significant differences were observed in ^18^F-FDG uptake between EGFR-mutated and EGFR wild type.
Lee S M et al.	206	SUVmax	^18^F-FDG avidity of NSCLC had no significant clinical value in predicting EGFR status.
Lee E Y et al.	71	pSUVmax, mSUVmax, and dSUVmax	No statistically significant difference was observed in SUVmax of the primary tumors and EGFR mutation status.
Du et al.	3574	SUVmax	SUVmax has low sensitivity and specificity in predicting EGFR mutations.
Guo et al.	4024	SUVmax, SUVmean	SUVmax and SUVmean had pooled sensitivity and specificity to predict EGFR mutation status.
Chung et al.	106	SUVmax, MTV, and TLG	No significant differences were found in FDG PET/CT parameters for EGFR mutation-negative and EGFR mutation-positive patients.
Cho et a	61	SUVmax, MTV, and TLG	SUVmax and TLG were significantly lower with EGFR mutation-positive lesions compared with EGFR wild type.
Liu et al.	82	SUVmax, MTV, TLG, clinicopathologic	Lower MTV combined with non-smokers and a peripheral tumor location were more likely to have EGFR mutations.
Yang et al.	200	SUVmax, SUVmean, MTV, and TLG	MTV demonstrated a significant difference between wild-type and mutant *EGFR* mutation status.
Liao et al.	191	SUVmax, MTV, TLG, CA199, and proGRP	Low MTV, proGRP, and female sex were independent significant predictors for EGFR mutation.

NSCLC, non-small cell lung cancer; SUV, standardized uptake value; MTV, metabolic tumor volume; TLG, total lesion glycolysis; CT, computed tomography; EGFR, epidermal growth factor receptor; TTF-1, thyroid transcription factor 1; CA199, carbohydrate antigen 199; proGRP, recombinant pro-Gastrin releasing peptide.

Na et al. evaluated the relationship between the EGFR mutation status and the SUVmax of ^18^F-FDG uptake by reviewing 100 patients with NSCLC (11), reporting that patients with a low SUVmax were more likely to have an EGFR mutation as compared to patients with a high SUVmax. Mak et al. (12) assessed 100 patients with NSCLC (24 EGFR mutants and 76 wild types), demonstrating that high FDG uptake in the primary tumor is related to a very low risk of an EGFR mutation. Subsequently, increasing evidence demonstrated that EGFR mutation status is associated with a lower SUVmax in NSCLC (9, 13). Chen et al. (14) showed that patients with an *EGFR* mutation showed decreased SUVmax values and subsequently reported that decreased FDG uptake associated with EGFR mutation status was *via* NOX4/ROS/GLUT1 axis. Yang et al. (15) analyzed 200 patients with lung adenocarcinoma, demonstrating that MTV of wild-type and mutant *EGFR* was significantly different. Furthermore, a study by Liao et al. (16) demonstrated that low primary MTV (pMTV) (<8.13 cm) was a strong and independent predictor and could be combined with female sex and gastrin-releasing peptide levels (proGRP, ≥38.44 pg/ml) to determine EGFR mutation status. In addition, decreased FDG uptake was shown to be a significant predictor of *EGFR* mutation status (17–22). Interestingly, EGFR mutation status was reported to be associated with a higher SUVmax (23, 24). Ko et al. (23) demonstrated a tendency of higher SUVmax in NSCLC patients with an EGFR mutation, and higher SUVmax could be combined with never smoking, carcinoma embryonic antigen (CEA) level, and a non-spiculated tumor margin to obtain a higher area under the receiver operating characteristic (ROC) curve for EGFR mutation status. A similar conclusion was reached by Kanmaz et al. (24).

However, multiple studies have shown no association between ^18^F-FDG uptake and EGFR mutation status. Chung et al. found no significant differences in ^18^F-FDG PET/CT parameters (SUVmax, MTV, and TLG) of EGFR mutation-positive and mutation-negative lung adenocarcinoma cases (25). Other studies confirmed that ^18^F-FDG metabolic parameters of PET/CT in NSCLC had no significant clinical value in predicting EGFR mutation status (26–29). The low diagnostic OR and the likelihood ratio scatter plot indicated that ^18^F-FDG PET/CT might be useless for predicting EGFR mutation status in NSCLC as indicated by a meta-analysis of Du et al. (30). According to a recent meta-analysis (31), SUVmax of the primary tumor had a moderate predictive value for EGFR mutation status in NSCLC. Due to this dispute, further high-quality studies are required to explore the predictive value of EGFR mutation status in NSCLC.

## 3 Predictive value of ^18^F-FDG PET/CT-derived radiomics with epidermal growth factor receptor mutation status in non-small cell lung cancer

**Figure 2:**
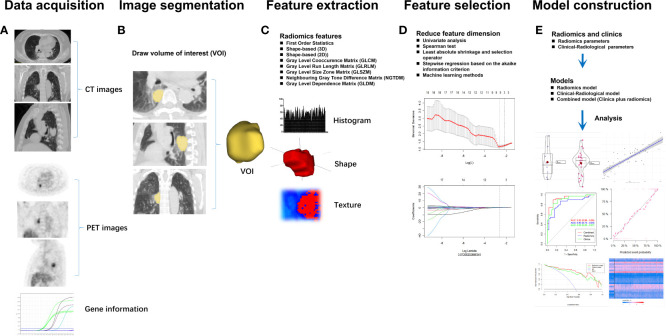
The workflow for radiomics analysis of ^18^F-FDG PET/CT data comprises five steps: **(A)** data acquisition, **(B)** image segmentation, **(C)** feature extraction, **(D)** feature selection, and **(E)** model construction.

Radiomics texture is an emerging field of interest in medical imaging and is a high-throughput and quantitative extraction of imaging features based on a computational approach (32). The rapid advance of emerging radiomics analysis could help discriminate the disease type, predict survival, and monitor the response to therapy using large datasets and artificial intelligence techniques (33). Radiomics also has various logistic advantages, for instance, offering nearly real-time results and being non-invasive (34). Additionally, compared with standard biopsy, radiomics can provide a comprehensive analysis of one lesion and multiple lesions within the examined area (35). The growing applications of ^18^F-FDG PET/CT radiomics have therefore attracted extensive interest in recent years, especially in lung cancer (36). The radiomics analysis of ^18^F-FDG PET/CT data comprises five steps: 1) data acquisition, 2) image segmentation, 3) feature extraction, 4) feature selection, and 5) model construction (**Figure 2**). Indeed, ^18^F-FDG PET/CT radiomics estimates of the tumor imaging phenotype extracted from PET/CT images facilitate the management of lung cancer, including differential diagnosis of benign/malignant solitary pulmonary nodules, NSCLC subtypes, lymph node metastasis, and distant metastases, as well as response evaluation and survival prediction (34, 37, 38). Increasing studies have confirmed the feasibility and potential superiority of ^18^F-FDG PET/CT radiomics to predict EGFR mutation status in NSCLC (**Table 2**).

**Table 2:** Recent publications about the predictive value of ^18^F-FDG PET/CT-derived radiomics with epidermal growth factor receptor mutation status in non-small cell lung cancer.

Authors	No. of patients	Aspect evaluated	Main results
Yip et al.	348	PET radiomics features	19 novel PET radiomics features were strongly associated with EGFR mutation status.
Park et al.	183	Heterogeneity of textural parameters of PET/CT	Heterogeneity textural parameters acquired from pretreatment FDG-PET/CT had clinical implications for identifying a high-risk subpopulation for EGFR TKI treatment.
Jiang et al.	80	PET and CT radiomics features	35 selected features were significantly associated with EGFR mutation status.
Koyasu et al.	138	Random forest (RF), gradient tree boosting (XGB)	In the classification of EGFR mutation status, the AUC values were as follows: RF, 0.625; XGB, 0.617.
Mu et al.	616	PET/CT-based deep learning model	Deep learning model to predict EGFR mutation status with AUCs of 0.86, 0.83, and 0.81 in the training, validation, and independent test cohorts, respectively.
Abdurixiti et al.	973	PET/CT-based radiomics	The ICC for summed RQS was 0.986 [95% confidence interval (CI): 0.898–0.998].
Yang et al.	174	PET/CT radiomics features,	The mutant/wild-type model was identified in the training (AUC, 0.77) and validation (AUC, 0.71) groups.
Zhang J et al.	248	PET/CT-based radiomics features	AUC is equal to 0.79 in the training set and 0.85 in the validation set, compared with 0.75 and 0.69 for the clinical model.
Zhang M et al.	173	PET/CT radiomics prediction model	Four CT and two PET radiomics features were finally selected to build the PET/CT radiomics model.
Shiri et al.	150	Low-dose CT, diagnostic CT, and PET radiomics	Multivariate machine learning-based AUC performances were significantly improved to 0.82 for EGFR.
Li et al.	115	PET/CT, CT radiomics features, conventional PET parameters	Wild-type of EGFR− cases with an AUC of 0.805, an accuracy of 80.798%, a sensitivity of 0.826, and a specificity of 0.783.
Chang et al.	583	PET/CT, CT, and PET radiomics models	The PET/CT radiomics–clinical combined model has better performance (AUC = 0.84) to predict EGFR mutation.

PET/CT, positron emission tomography/computed tomography; EGFR, epidermal growth factor receptor; NSCLC, non-small cell lung cancer.

To our knowledge, studies demonstrating the relationship between ^18^F-FDG PET/CT imaging textures and EGFR mutation status are limited. However, they have proved that prediction models based on ^18^F-FDG PET/CT imaging features can help differentiate EGFR mutation status in NSCLC, which is crucial in clinical practice to identify candidates for targeted therapy (39–44). Yang et al. (45) used ^18^F-FDG PET/CT-based radiomics features integrated with clinical features and ^18^F-FDG PET/CT metabolic parameters (MTV, TLG, SUVmax, and SUVmean) of 174 lung adenocarcinoma patients to establish prediction models and achieved an area under the curve (AUC) of 0.71–0.77. Shiri et al. (46), Zhang et al. (47), and Zhang et al. (48) reached a similar conclusion.

Li et al. (49) showed that radiomics signatures derived from ^18^F-FDG PET/CT images were significantly more predictive of EGFR mutations than those derived from CT or conventional PET images. In addition, a recent study found that PET/CT radiomics model has a better capability (AUC = 0.76) to predict EGFR mutation status than the PET radiomics model (AUC = 0.71) and the CT radiomics model (AUC = 0.74) in NSCLC (50). A meta-analysis by Abdurixiti et al. (51) revealed that PET/CT-based radiomics signatures could be used as a diagnostic index for EGFR mutation status in patients with NSCLC.

The reachable results in the literature are definitely promising; ^18^F-FDG PET/CT-based radiomics has the potential to replace classic approaches based on biopsy and histopathology to detect EGFR mutation status in NSCLC. However, the results should be interpreted with caution, as there is a lack of reproducibility and a basic deficiency of normalization methods and settings (52), so further studies are essential to establish a consistent approach. Furthermore, a high-quality predictive model depends on a large amount of data, so additional studies involving larger multicenter cohorts will be needed to develop this method into a clinical tool.

## 4 A new type of molecular PET/CT probe to evaluate epidermal growth factor receptor mutation status in non-small cell lung cancer

^18^F-FDG metabolic parameters associated with EGFR mutation status in NSCLC reflect the tumor cell glucose metabolism of tumor cells, which have poor sensitivity and are limited by many factors. Therefore, the targeting moiety or ligand must be attached with an applicable labeling agent for the imaging modality to accurately evaluate EGFR mutation status or guide EGFR-TKI treatment. Antibodies are often used due to their sufficient high-affinity specific EGFR (wild and mutated) binding. Currently, the molecular imaging modalities employed for detecting EGFR mutations are SPECT, PET, and PET/CT. Isotopic labeling substances may be combined with monoclonal antibodies to EGFR or EGFR-TKI molecular probes to reflect EGFR mutation status according to radioactive uptake in PET/CT images. Previous studies mainly used radioactive nuclides such as ^86^Y, ^64^Cu, and ^89^Zr to label anti-EGFR monoclonal antibodies (including cetuximab and panitumumab) and ^11^C and ^18^F to label EGFR-TKI (involved PD153035, gefitinib, erlotinib, and afatinib). However, current research focuses on cell and animal experiments with little clinical application (**Table 3**).

**Table 3:** Recent publications about the new type of molecular probe of PET/CT in use for the detection of epidermal growth factor receptor mutation status in non-small cell lung cancer.

Authors	No. of patients	New type of molecular probe	Main results
Lui et al.	11	^11^C-PD153035	EGFR expression in NSCLC primary tumors with ^11^C-PD153035 uptake, and the SUVs were also correlated with the EGFR expression level.
Meng et al.	21	^11^C-PD153035	^11^C-PD153035 uptake is close to the EGFR expression level in NSCLC.
Sun et al.	75	^18^F-MPG	^18^F-MPG uptake is significantly accelerated in NSCLC tumors harboring EGFR-activating mutations.
Van Loon et al.	6	^89^Zr-cetuximab	No direct significant association was found between SUVmax, SUVmean, and EGFR IHC score.
Memon et al.	30	^11^C-Erlotinib	Variation in ^11^C-erlotinib accumulation between different malignant lesions in the same patient.
Bahce et al.	10	^11^C-Erlotinib	^11^C-Erlotinib accumulated in tumors that expressed high levels of EGFR and were sensitive to TKI therapy.
Bahce et al.	10	^11^C-Erlotinib	Tumor ^11^C-erlotinib uptake in NSCLC patients after erlotinib therapy was reduced and further illustrated the ^11^C-erlotinib binding specificity of EGFR mutation.
Song et al.	3	^18^F-IRS	PET/CT imaging with ^18^F-IRS showed a potential to diagnose NSCLC EGFR mutation.
Stadt et al.	10	^18^F-Afatinib	^18^F-Afatinib can potentially be used in evaluating EGFR mutation-positive patients.
Stadt et al.	12	^18^F-Afatinib	^18^F-Afatinib PET/CT could provide methods to identify EGFR mutation-positive patients who benefit from afatinib therapy.

^11^C-PD153035, ^11^C-labeled 4-N-(3-bromoanilino)-6,7-dimethoxyquinazoline; ^18^F-MPG, ^18^F-labeled2-(2-(2-(2-(4-(3-chloro-4-fluorophenylamino)-6-methoxyquinazolin-7-yl)oxy)ethoxy)ethoxy)ethoxy)ethyl 4-methylbenzenesulfonate; ^18^F-IRS, ^18^F-N-(3-chloro-4-fluorophenyl)-7-(2(2-(2-(2-(4-fluorine)ethoxy)ethoxy)-ethoxy)-6-(3-morpholinopropoxy)quinazoline-4-amine.

### 4.1 Monoclonal antibody probes

Monoclonal antibodies directly target the extracellular domain of EGFR to prevent the binding of EGFR to ligands, thus blocking downstream signal transduction pathways. Monoclonal antibodies are all large molecules that need to be labeled with radionuclides with a long half-life, such as ^64^Cu, ^11^C, and ^89^Zr, as they infiltrate tissue very slowly. PET/CT using ^89^Zr-cetuximab allowed the visualization and quantification of tumor ^89^Zr-cetuximab uptake in cells and animals (53) or other malignancies (54) with EGFR mutations. Van Loon et al. studied head and neck cancer (NHC) and NSCLC patients using ^89^Zr-cetuximab PET/CT but showed that SUVmax and SUVmean had no direct relationship between EGFR immunohistochemistry (IHC) score and tumor-to-background ratio (TBR) (55). ^89^Zr-DFO-panitumumab PET/CT imaging assessed EGFR expression at a cellular level and in animals (56, 57).

### 4.2 Epidermal growth factor receptor–tyrosine kinase inhibitors molecular probes

Radiolabeled EGFR-TKI can bind specifically to the tyrosine kinase domain of the mutant protein, and the uptake levels can reflect EGFR expression and mutation status. Therefore, EGFR-TKI molecular probes have many obvious advantages over monoclonal antibodies. EGFR-TKI molecular probes are labeled with radionuclides of short circulating half-life, such as ^11^C and ^18^F, which can penetrate tissues quickly because they are small molecules.

#### 4.2.1 ^11^C-PD153035

4-*N*-[3-bromoanilino]-6,7-dimethoxyquinazoline (PD153035) is a reversible inhibitor of EGFR tyrosine kinase and a potent ATP-competitive TKI of EGFR (58). Additionally, ^11^C-labeled PD153035 has been assessed *in vivo* as a PET/CT agent to estimate EGFR expression in multiple tumors (59). Liu et al. studied the distribution of ^11^C-PD153035 in PET/CT imaging of 11 patients with NSCLC, finding that SUVs were correlated with expression levels of EGFR (60). Meng et al. analyzed ^11^C-PD153035 PET/CT images of 21 NSCLC patients revealing that ^11^C-PD153035 uptake is closely related to EGFR expression (61). Dai et al. demonstrated that ^11^C-PD153035 PET/CT imaging can be used as a simple and efficient method to detect NSCLC patients who are sensitive to EGFR-TKIs (62). Furthermore, the synthesis of polyethylene glycol (PEG)-modified (PEGylated) anilinoquinazoline derivative, 2-(2-(2-(2-(4-(3-chloro-4-fluorophenylamino)-6-methoxyquinazolin-7 yl)oxy)ethoxy)ethoxy)ethoxy)ethyl 4-methylbenzenesulfonate (T-MPG) derived from the known EGFR-TKI PD153035 has been reported by Sun et al. (63). Not only their preclinical research but also clinical research that involved 75 NSCLC patients has suggested that ^18^F-MPG uptake is dramatically accelerated in *EGFR*-mutated NSCLC.

#### 4.2.2 ^11^C-Erlotinib

^11^C-Erlotinib is a PET imaging tracer with great promise for evaluating EGFR expression in NSCLC patients and has been reported in animal models and human subjects, but only a limited number of clinical PET/CT studies have been conducted. Bahce et al. illustrated that ^11^C-erlotinib accumulated in tumors that highly expressed EGFR by reviewing ^11^C-erlotinib PET/CT images of 10 patients with NSCLC (64). A study by Bachce et al. analyzed 10 NSCLC patients with EGFR mutation status, demonstrating that ^11^C-erlotinib uptake in tumors reduces after erlotinib therapy (65). However, Petrulli et al. showed a lack of association between EGFR mutation status and ^11^C-erlotinib uptake in an analysis of 10 NSCLC patients *via* dynamic multi-bed PET/CT scan using ^11^C-erlotinib, suggesting disease heterogeneity and low tracer uptake for the lack of association (66).

#### 4.2.3 ^11^C-/^18^F-Gefitinib

Gefitinib is a small-molecule EGFR kinase inhibitor that binds to the intracellular tyrosine kinase domain and disrupts EGFR kinase activity with nanomolar affinity (67). ^11^C- and ^18^F-radiolabeled gefitinib could be applied to image EGFR expression and pharmacokinetics non-invasive study of gefitinib in patients. However, a few studies have been conducted at the cell and animal levels, and human tumor xenografts have not shown EGFR-specific concentrations (68). However, a novel radiotracer, ^18^F-*N*-(3-chloro-4-fluorophenyl)-7-(2(2-(2-(2-(4-fluorine)ethoxy)ethoxy)-ethoxy)-6-(3-morpholinopropoxy)quinazoline-4-amine (^18^F-IRS) based on gefitinib has been designed and synthesized, with ^18^F-IRS PET/CT showing potential to diagnose NSCLC EGFR mutation according to higher ^18^F-IRS uptake in NSCLC with EGFR mutations (69).

#### 4.2.4 ^18^F-Afatinib

Afatinib is a second-generation irreversible 4-anilinoquinazoline EGFR kinase inhibitor (70). In mouse models bearing NSCLC xenografts [EGFR-mutated (HCC827 and H1975) xenografts and EGFR wild-type (A549)], Slobbe et al. suggested accumulation of ^18^F-afatinib in NSCLC tumors with EGFR mutation status (71, 72), justifying the further evaluation of NSCLC tumor EGFR mutations. Stadt et al. (73) quantified ^18^F-afatinib tumor uptake in NSCLC patients, suggesting that ^18^F-afatinib could potentially be used to evaluate EGFR mutation-positive patients. Furthermore, Stadt et al. (74) also evaluated whether ^18^F-afatinib uptake could predict the response to afatinib therapy by evaluating ^18^F-afatinib PET/CT images of 12 patients with NSCLC, showing that ^18^F-afatinib PET/CT could serve as a method for precise quantification of EGFR mutation status in NSCLC patients who would benefit from afatinib therapy.

The possibilities of protein molecular probes targeting EGFR have been demonstrated in *in vivo* imaging cell, animal, and clinical studies, especially EGFR-TKI-type molecular probes. Although these studies showed that molecular probes targeting EGFR for PET/CT imaging can identify EGFR mutation status in NSCLC, they tend to produce high background noise because of high lipophilicity, which leads to poor imaging quality. The short half-life of ^11^C also limits its widespread use in clinical practice, and ^18^F labeling requires many procedures to label the TKIs.

## 5 Conclusion

EGFR is a significant target for lung cancer diagnosis and treatment; thus, non-invasive, accurate, and rapid methods for EGFR mutation detection should be developed in NSCLC. Due to recent advances in molecular imaging and analytic platforms, PET/CT may play a crucial role in identifying EGFR mutation status. The relatively new ^18^F-FDG PET/CT-derived radiomics to predict EGFR mutations has attracted much attention, with studies revealing promising results. PET/CT imaging with radiolabeled monoclonal antibodies and EGFR TKIs is particularly attractive and may be better than ^18^F-FDG PET/CT-derived radiomics in detecting EGFR mutation status in NSCLC because it can be repeatedly operate and reflect receptor status in real-time. However, since most of the research to date has been performed at the cellular level or in animals, further clinical studies are needed in the future.

## Author contributions

Conceptualization: NH, PGL, and PL. Writing (original draft preparation): NH, GY, YHW, and PGL. Writing (review and editing): PGL, YW, LW, and YNX. All the authors have read the manuscript and have approved it before submission.

## Funding

This work was supported partly by the Science and Technology Projects of Guizhou Province (Qiankehe Support [2020]4Y193, Qiankehe Basic-ZK[2022]General 422) and the National Natural Science Foundation of China (81960338).

## Acknowledgments

The authors gratefully thank all the participants at Guizhou Medical University. They are also thankful to StudyForBetter Team who contributed their best research skills to the area of radiobioinformatics.

## Conflict of interest

The authors declare that the research was conducted in the absence of any commercial or financial relationships that could be construed as a potential conflict of interest.

## Publisher’s note

All claims expressed in this article are solely those of the authors and do not necessarily represent those of their affiliated organizations, or those of the publisher, the editors and the reviewers. Any product that may be evaluated in this article, or claim that may be made by its manufacturer, is not guaranteed or endorsed by the publisher.
